# Development of lotus seed-based vegan yogurt: fermentation optimization, quality evaluation and analysis of key bioactive alkaloids

**DOI:** 10.3389/fnut.2026.1825059

**Published:** 2026-04-28

**Authors:** Mingyue Chi, Wenli Wu, Yiyuan Wu, Jiamiao Hu, Baodong Zheng, Shaoxiao Zeng, Shaoling Lin

**Affiliations:** College of Food Science, Fujian Agriculture and Forestry University, Fuzhou, Fujian, China

**Keywords:** bioactive alkaloids, fermentation, lotus seeds, plant-based yogurt, sleep-promoting

## Abstract

**Introduction:**

Lotus seed is a nutritious and functional food material, yet its utilization in plant-based fermented products remains underdeveloped. This study aimed to develop a novel plant-based yogurt using lotus seeds as the primary raw material, and to systematically optimize the probiotic strain combination and fermentation process to improve product quality and functionality.

**Methods:**

The optimal lactic acid bacteria strain combination and key fermentation parameters (temperature, time, glucose addition, and inoculum size) were determined through single-factor experiments and response surface methodology.

**Results:**

The optimal strain combination was *Lactobacillus delbrueckii* subsp. *bulgaricus, Lactiplantibacillus plantarum*, and *Streptococcus thermophilus* at a ratio of 1:2:1. The optimized product, fermented at 38 °C for 7.5 h with 12.2% glucose and a 3.7% inoculum, exhibited high water-holding capacity (68.57%) and favorable sensory acceptance (80.9 points). The yogurt presented a uniform white color, smooth texture, and characteristic lotus seed aroma. Nutritional analysis showed significantly lower fat and calorie content, along with higher dietary fiber, compared to conventional dairy yogurt. Microstructural observations indicated that fermentation promoted the formation of a uniform and compact gel network, enhancing texture and stability. Notably, fermentation effectively preserved key bioactive alkaloids from lotus seeds, including neferine and nuciferine.

**Discussion:**

This study successfully developed a plant-based yogurt from lotus seeds with desirable physicochemical, sensory, and nutritional properties. The optimized fermentation process also retained important bioactive compounds, offering a theoretical basis and practical guidance for the value-added utilization of lotus seeds and the development of functional plant-based fermented foods with potential health benefits.

## 1 Introduction

Plant-based vegan yogurt is a fermented food derived from various plant substrates that exhibits a texture and sensory profile comparable to those of conventional dairy yogurt (1). Similar to dairy-based counterparts, plant-based yogurts are capable of maintaining viable lactic acid bacteria throughout extended storage periods (2) and demonstrate improved flavor and mouthfeel, as well as enhanced functional attributes (3). With increasing consumer awareness of healthy dietary patterns, plant-based yogurts have gained considerable attention as suitable alternatives for lactose-intolerant individuals, vegans, and other special population groups (4, 5). Consequently, global market demand for plant-based yogurt products continues to grow steadily (6). However, the development of plant-based yogurts derived from high-starch botanical sources remains challenging, as high starch content during fermentation often leads to weak gel formation, pronounced syneresis (whey separation), and insufficient fermentation activity (7). Among common high-starch ingredients such as corn, cassava, and oats, lotus seed (*Nelumbo nucifera* Gaertn.) presents a uniquely challenging set of technical hurdles. The amylose content of lotus seed starch is considerably higher than that of typical high-starch plants, making it highly prone to rapid retrogradation after gelatinization (8). This manifests as pronounced syneresis and textural hardening, which severely compromise the gel stability of plant-based yogurt during fermentation and cold storage. Additionally, lotus seed starch has a high gelatinization peak temperature of 72–79 °C and exhibits low swelling power, limiting its ability to fully gelatinize and hindering the formation of a stable gel network (9). Taken together, these factors make the development of lotus seed-based plant yogurt particularly difficult, positioning lotus seed as a quintessential example of a challenging, high-starch ingredient in plant-based yogurt formulation.

Lotus seeds are regarded as a quintessential example of a substance with “food-medicine homology.” Originating from traditional Chinese medicine, this concept refers to edible substances that serve not only as a source of daily nutrition but also as functional components with physiological effects that support health maintenance (10). Although this notion is deeply rooted in traditional medicine, its relevance to modern food science lies in the scientifically validated bioactivities of the constituents found in lotus seeds. Lotus seeds are rich in starch and protein (11) and contain a variety of bioactive compounds, including alkaloids and polyphenols (12). Among these, certain alkaloids—such as nuciferine—have been scientifically validated for their sleep-promoting effects (13), highlighting the potential of lotus seeds as functional ingredients for sleep-related dietary interventions. However, the direct consumption of lotus seeds presents several limitations. Their dense starch granule structure may increase digestive burden, while their astringent characteristics can cause gastrointestinal discomfort (14). Notably, lactic acid bacteria fermentation may provide an effective strategy to overcome these limitations and enhance the nutritional and physiological value of lotus seeds by producing beneficial metabolites (15). Consequently, yogurt fermentation may not only alleviate the adverse effects associated with direct lotus seed consumption, but also facilitate the biotransformation of lotus seed components, thereby improving their bioavailability and functional properties. This approach aligns closely with the core principles of food-medicine homology, leveraging modern food processing techniques to transform a traditionally recognized health-promoting ingredient into a more digestible and bioavailable functional food matrix.

Therefore, the present study aimed to develop a novel plant-based yogurt using lotus seeds as the main raw material, with the goal of achieving a smooth texture, desirable flavor, and good storage stability. Lactic acid bacteria strains were systematically screened, and fermentation conditions were optimized through single-factor experiments and response surface methodology. The resulting product was comprehensively characterized in terms of nutritional composition, rheological properties, flavor evolution during fermentation, microstructural features, and refrigerated storage stability. We hypothesize that optimizing fermentation parameters and probiotic strains will lead to a lotus seed-based yogurt with desirable physicochemical, sensory, and nutritional properties, while also preserving or enhancing bioactive alkaloids. To test this hypothesis, several alkaloids in the finished product were quantitatively analyzed, providing a compositional basis for its potential sleep-aid functionality.

Overall, this study offers a novel plant-based yogurt product that aligns with consumer demand for healthy and functional foods, while also promoting the high-value utilization of lotus seeds. The findings provide both scientific and practical insights into the development of functional plant-based fermented foods and contribute to the diversification and value enhancement of the plant-based food industry.

## 2 Materials and methods

### 2.1 Materials

Frozen fresh lotus seeds were purchased from Fuzhou Shengdian Food Co., Ltd. (Fuzhou, China), as detailed in Supplementary Table S1. *Lactobacillus helveticus* LH76, *Lactobacillus delbrueckii* subsp. *bulgaricus* LB42, *Lactiplantibacillus plantarum* Lp90, *Lactobacillus acidophilus* LA85, *Lacticaseibacillus rhamnosus* LRa05, and *Streptococcus thermophilus* ST81 were obtained from Weikang Probiotics (Suzhou) Co., Ltd. (Suzhou, China). MRS agar and MRS broth medium were purchased from Guangdong Huankai Microbial Science & Technology Co., Ltd. (Guangdong, China). Sodium chloride, sodium hydroxide, phenolphthalein, and absolute ethanol were purchased from China National Medicines Corporation Ltd. (Beijing, China). Glucose was supplied by Fuzhou Haiwang Fuyo Pharmaceutical Co., Ltd. (Fuzhou, China). A commercial dairy yogurt (Classy-Kiss) was purchased from Yonghui Supermarket (Fuzhou, China). The product was formulated with raw milk as the primary ingredient, constituting ≥ 80% (w/w).

### 2.2 Preparation of plant-based lotus seed yogurt

First, the frozen lotus seeds (with embryos removed) were thawed, rinsed and blended with purified water at 1:3 (w/v) to obtain a smooth slurry. Glucose was then added to the slurry, and the mixture was filled into containers, hermetically sealed, and pasteurized at 95 °C for 15 min. After cooling, the sterile slurry was inoculated with the lactic acid bacteria strain and fermented at a controlled temperature. The resulting yogurt was finally cooled to 4 °C and held for 24 h to complete post-ripening, yielding the finished yogurt.

For lactic acid bacteria strain optimization, *L. helveticus, L. bulgaricus, L. plantarum, L. acidophilus, L. rhamnosus* and *S. thermophilus* were individually activated in MRS broth through three successive subcultures at 37 °C for 48 h. Each strain was inoculated into lotus seed slurry at 4% (v/v), resulting in an initial viable count of approximately 4 × 106 CFU/g, and fermented at 37 °C for 12 h. Fermented samples were evaluated for water-holding capacity (WHC), pH, acidity, and viable counts according to Zhao et al. (16). Next, three optimal strains (*L. bulgaricus, L. plantarum, S. thermophilus*) were selected and combined in ratios of 2:1:1, 1:2:1, and 1:1:2. This design was based on the established principle that a dominant strain inevitably emerges in mixed lactic acid bacteria strains (17). In these ratios, the dominant strain was set to comprise 50% of the inoculum, with each auxiliary strain accounting for 25%, in accordance with the approach reported by Barrette et al. (18). A constant total inoculum size was maintained across all formulations to ensure comparability. Fermentation was conducted under the same conditions and evaluated as described above.

### 2.3 Optimization of preparation process for plant-based lotus seed yogurt

#### 2.3.1 Single-factor experiments

Based on the fermentation process outlined in Section 2.2, single-factor experiments were conducted to evaluate the effects of glucose addition (6%, 9%, 12%, 15%, m/m), inoculum size (2%, 4%, 6%, v/v), fermentation temperature (36 °C, 38 °C, 40 °C, 42 °C), and fermentation time (6 h, 8 h, 10 h, 12 h). Sensory score was used as the evaluation criterion.

#### 2.3.2 Response surface methodology experiment

Based on the results obtained from single-factor experiments, a four-factor, three-level Box-Behnken design was established using Design-Expert 12 software (19). The independent variables were glucose addition (A), inoculum size (B), fermentation temperature (C), and fermentation time (D). WHC (Y_1_) and sensory score (Y_2_) were selected as response variables. The experimental design is presented in Table 1. The experimental data were fitted to a second-order polynomial model as follows:


$$\begin{array}{l} Y=\beta_{0}+\overset{4}{\underset{i=1}{\sum }}\beta_{i}X_{i}+\overset{4}{\underset{i=1}{\sum }}\beta_{ii}X_{i}^{2}+\overset{4}{\underset{i=1}{\sum }}\overset{4}{\underset{j=i+1}{\sum }}\beta_{ij}X_{i}X_{j}+\varepsilon \end{array}$$ (1)

**Table 1:** Box-behnken test factors and levels.

Levels	Factors			
	A: Glucose addition (%)	B: Inoculum size (%)	C: Fermentation temperature (°C)	D: Fermentation time (h)
−1	10	3	37	7
0	12	4	38	8
1	14	5	39	9

#### 2.3.3 Sensory evaluation of plant-based lotus seed yogurt

Sensory evaluation was conducted using the quantitative descriptive analysis (QDA) method, with reference to the description by Talens et al. (20) and with slight modifications. Sensory evaluation was conducted by a panel of 10 assessors under controlled conditions (22 ± 2 °C). All panelists had at least one year of experience in evaluating dairy or plant-based products. Prior to formal evaluation, a calibration session was held to familiarize the panelists with the evaluation criteria and to standardize scoring using reference samples. Samples were presented in a randomized, blind-coded order and evaluated for texture (30 points), mouthfeel (30 points), aroma (20 points), and color (20 points), as shown in Table 2, with higher weights assigned to texture and mouthfeel (30 points each) as these attributes are widely recognized as the most critical factors influencing product stability and consumer perception in plant-based fermented products (3).

**Table 2:** Sensory evaluation scale.

Item	Scoring criteria
Texture (30 points)	Good coagulation state, moderate thickness, stringy texture, no bubbles, no whey separation (21–30)
	Moderate coagulation state, appropriate thickness, few bubbles and slight whey separation (11–20)
	Poor coagulation state, grainy, surface cracks and bubbles, obvious whey separation (0–10)
Mouthfeel (30 points)	Sweet and sour taste, with pleasant lotus seed aroma (21–30)
	Moderately sweet and sour, weak lotus seed flavor (11–20)
	Overly sour or sweet, uncoordinated flavor (0–10)
Aroma (20 points)	Rich and harmonious lotus seed aroma, no off-flavors (14–20)
	Light lotus seed aroma, no off-flavors (8–13)
	Bland lotus seed aroma, with off-flavors (0–7)
Color (20 points)	Uniform color, milky white, glossy (14–20)
	Relatively uniform color, light yellow, slightly less glossy (8–13)
	Non-uniform color, slightly yellow, poor gloss (0–7)

### 2.4 Analysis of nutritional components and quality of plant-based lotus seed yogurt

#### 2.4.1 Determination of nutritional components

The nutritional composition of the plant-based lotus seed yogurt was determined and compared with that of a commercial dairy yogurt (Classy-Kiss). Protein, fat, and moisture contents were analyzed according to AOAC methods (21). Carbohydrates were determined from the difference in the nutrients according to Legislative Decree n. 77 of 16 February 1993, and energy value was calculated using standard conversion factors (9.0 kcal/g for fat, and 4.0 kcal/g for protein and carbohydrate) (22). Starch content was determined following McCleary et al. (23), and dietary fiber was measured using AOAC method 985.29.

#### 2.4.2 Determination of physicochemical and microbiological indicators

*E. coli* counts were determined according to ISO 7251:2005 (24). Samples were diluted and inoculated into LST broth (37 °C), then positive tubes were transferred to EC broth (44 °C) and confirmed by indole test. The detection limit was 3 MPN/g. Viscosity was measured using an Alpha Re rotational viscometer, and color parameters (L^*^, a^*^, b^*^) were recorded using ADCLP fully automatic colorimeter.

#### 2.4.3 Determination of rheological properties

Rheological measurements of unfermented lotus seed slurry, plant-based lotus seed yogurt, and commercial dairy yogurt were conducted using an MCR 3031 rheometer (Anton Paar, Austria) following Devnani et al. (25) with minor modifications. A 40-mm parallel plate geometry with a 1-mm gap was used at a constant temperature of 25 °C. Apparent viscosity was evaluated through a shear rate sweep from 0.1 to 100 s^−1^. Viscoelastic properties were characterized by frequency sweep measurements at a fixed strain of 1% over an angular frequency range of 0.1–10 rad/s, from which the storage modulus (G′) and loss modulus (G″) were determined.

#### 2.4.4 Flavor component analysis

Volatile compounds were analyzed using a PEN3.5 electronic nose (AIRSENSE GmbH, Germany) following a method of Yuan et al. (26) with slight modifications. For each measurement, 5 mL of sample was placed in a 20 mL headspace vial and incubated at 50 °C for 30 min prior to analysis.

Taste profiles were evaluated with an SA402B electronic tongue (INSENT Co., Japan) following a method of Yuan et al. (26) with slight modifications. Sample preparation involved diluting 10 mL of the yogurt 90-fold with distilled water before measurement.

#### 2.4.5 Microstructural analysis

Scanning Electron Microscopy (SEM): Microstructural analysis was performed using a Sigma 300 scanning electron microscope (ZEISS, Germany), following the method described by Li et al. (27) with minor modifications. Samples were freeze-dried and gold-coated prior to observation. Observations were conducted at an accelerating voltage of 30 kV to examine the microstructure of both plant-based lotus seed yogurt and unfermented lotus seed slurry.

FTIR Analysis: The short-range ordered structures of the plant-based lotus seed yogurt and unfermented lotus seed slurry were analyzed by FTIR spectroscopy, following the method described by Li et al. (27) with minor modifications. Briefly, the freeze-dried sample powder was mixed with potassium bromide (KBr) at a ratio of 1:100 (w/w), thoroughly ground, and pressed into a transparent pellet. FTIR spectra were collected at a resolution of 4 cm^−1^ over the wavenumber range of 400–4000 cm^−1^.

#### 2.4.6 Storage period experiment

Storage stability was evaluated during 31 days of refrigerated storage (4 °C). Changes in pH, WHC, and viable probiotic counts were monitored at 5-day intervals.

### 2.5 Analysis of alkaloids by HPLC-MS/MS

#### 2.5.1 Sample preparation

Alkaloids were extracted from yogurt samples using ultrasound-assisted ethanol extraction as described by Shi et al. (28) using ultrasound-assisted extraction method with ethanol as the solvent. Extracts were centrifuged and filtered through a 0.22 μm membrane prior to analysis.

#### 2.5.2 Chromatographic conditions

The analytical separation was performed using an ExionLC UPLC system. The column was equilibrated with a binary gradient consisting of Mobile Phase A (MPA; water with 0.1% (v/v) formic acid) and Mobile Phase B (MPB; acetonitrile) under the initial conditions of 95% MPA and 5% MPB. The flow rate was set at 0.80 mL/min, and the column temperature was maintained at 40 °C throughout the analysis. A linear gradient elution program was applied as follows: 0–4.0 min, 5% MPB; 4.0–6.0 min, 80% MPB; 6.0–6.1 min, 5% MPB; 6.1–8.0 min, 5% MPB. Detection was conducted in positive ion mode using MRM on a QTRAP 4,500 mass spectrometer. The total chromatographic run time was 8 min.

#### 2.5.3 Identification and quantification

Target alkaloids were identified by matching retention times and characteristic MRM transitions with authentic standards. Quantification was performed using the external standard method. Calibration curves were constructed using quadratic regression, and alkaloid concentrations were calculated from the corresponding calibration equations (Tables 3, 4).

**Table 3:** MS parameters for the target alkaloids in MRM mode.

Compound	Q1 mass (m/z)	Q3 mass (m/z)	DP(V)	CE(V)	Purpose
Liensinine	611.3	206.1	13	45	Quantification
Liensinine	611.3	489.3	13	46	Qualification
Liensinine	611.3	580.4	13	45	Qualification
Neferine	625.3	206.2	4	46	Quantification
Neferine	625.3	489.3	4	49	Qualification
Neferine	625.3	594.3	4	46	Qualification
Nuciferine	295.6	235.1	19	46	Qualification
Nuciferine	295.6	219.1	19	47	Qualification
Nuciferine	295.6	250.2	19	31	Qualification
Isococlaurine	285.6	106.9	59	40	Qualification
Isococlaurine	285.6	115.0	59	59	Qualification
Isococlaurine	285.6	194.0	59	53	Qualification
Armepavine	315.6	107.0	69	34	Qualification
Armepavine	315.6	238.0	69	50	Qualification
Armepavine	315.6	108.1	69	43	Qualification

DP, Declustering Potential; CE, collision energy.

**Table 4:** Retention times and calibration curve parameters for the five alkaloids.

Compound	Retention time (min)	Linear range (μg/L)	Regression equation	R^2^
Liensinine	3.21	1 μg/L−100 μg/L	y = 6.23936x^2^+5475.77489x−694.86963	0.9999
Neferine	3.40	1 μg/L−100 μg/L	y = −5.52309x^2^+8896.30811x−5108.38285	0.9999
Nuciferine	3.98	1 μg/L−100 μg/L	y = −5.00270x^2^+5875.51278x+369.60093	1.0000
Isococlaurine	3.24	5 μg/L−400 μg/L	y = −1.65304x^2^+3477.92356x+3500.08946	0.9999
Armepavine	3.45	5 μg/L−400 μg/L	y = −0.06608x^2^+241.04053x+283.01401	0.9999

### 2.6 Statistical analysis

All experiments were conducted in triplicate, and results are expressed as mean ± SD. Statistical analysis was performed using SPSS 25 with one-way analysis of variance (ANOVA) followed by Tukey's honestly significant difference (HSD) *post-hoc* test for multiple comparisons. Significance was defined at *p* < 0.05. Figures were generated using Origin 2024.

## 3 Results

### 3.1 Screening of optimal strains and inoculation ratios

The six strains exhibited distinct fermentation performances (Table 5). *L. bulgaricus* and *L. plantarum* demonstrated superior WHC, viable counts, and acidity. *L. helveticu*s and *L. rhamnosus* showed poor overall performance, while *S. thermophilus* and *L. acidophilus* exhibited comparable fermentation behavior. Based on these results, *L. bulgaricus, L. plantarum*, and *S. thermophilus* were selected as the optimal strains for subsequent mixed-culture fermentation.

**Table 5:** WHC, pH, acidity, and viable cell count of six strains after individual fermentation.

Fermentation strain	WHC/%	pH	Acidity/°T	Viable count/10^8^CFU/g
*L. helveticus*	54.08 ± 0.26^b^	3.69 ± 0.03^c^	80.90 ± 0.53^b^	0.74 ± 0.09^c^
*L. bulgaricus*	58.17 ± 0.25^a^	3.84 ± 0.03^a^	72.27 ± 0.31^d^	1.36 ± 0.06^a^
*L. plantarum*	57.98 ± 0.57^a^	3.80 ± 0.01^b^	71.60 ± 0.40^d^	1.25 ± 0.04^ab^
*L. acidophilus*	52.16 ± 0.31^c^	3.71 ± 0.03^c^	79.40 ± 0.35^c^	1.13 ± 0.09^b^
*L. rhamnosus*	52.34 ± 0.32^c^	3.72 ± 0.02^c^	88.43 ± 0.60^a^	0.72 ± 0.18^c^
*S. thermophilus*	54.38 ± 0.43^b^	3.71 ± 0.01^c^	80.33 ± 0.31^b^	1.15 ± 0.08^b^

Different lowercase letters in the same column indicate significant differences (*p* < 0.05).

**Figure 1:**
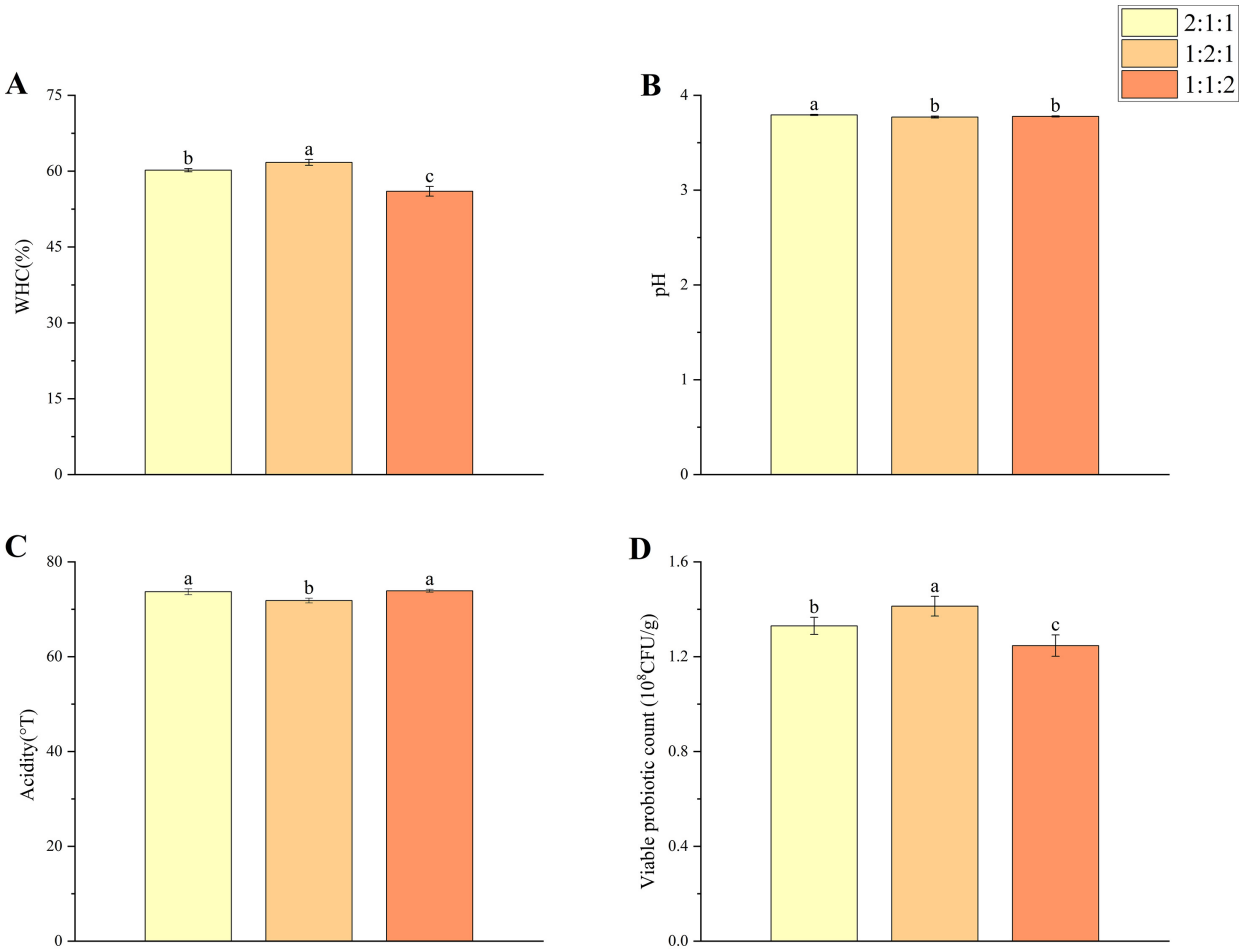
Effects of different ratios of *L. bulgaricus, L. plantarum*, and *S. thermophilus* on the physicochemical properties of plant-based lotus seed yogurt: **(A)** WHC, **(B)** pH, **(C)** acidity, and **(D)** viable probiotic count. Different letters indicate significant differences (*p* < 0.05).

Three mixed-culture ratios of *L. bulgaricus, L. plantarum*, and *S. thermophilus* (2:1:1, 1:2:1, and 1:1:2) were evaluated (Figure 1). All mixed cultures outperformed single-strain fermentations, suggesting the benefits of microbial synergy. Among them, the 1:2:1 ratio achieved the highest WHC and viable counts; while the other two groups showed inferior performance.

### 3.2 Optimization of fermentation parameters for lotus seed-based vegan yogurt

**Figure 2:**
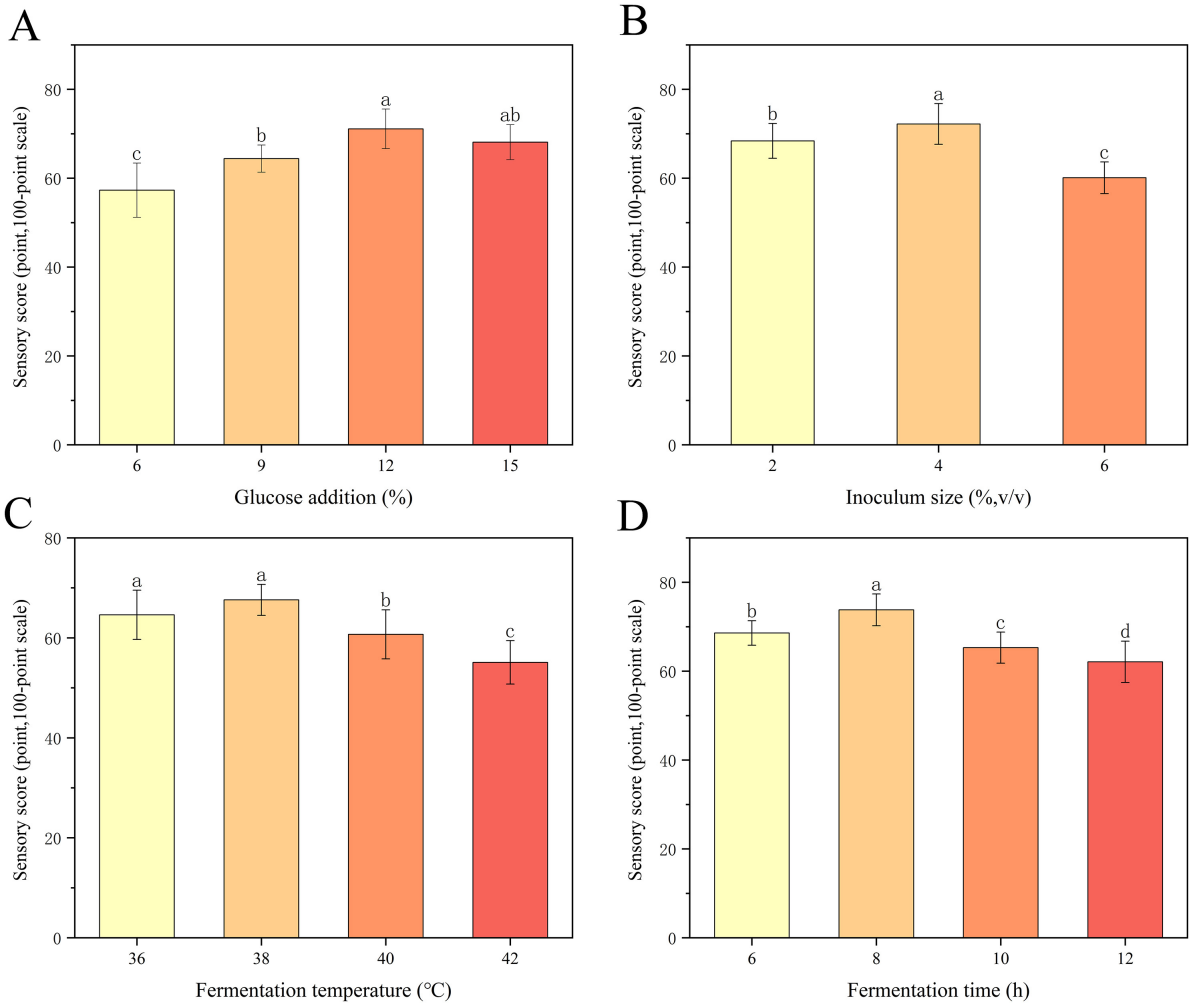
Effects of **(A)** glucose addition, **(B)** inoculum size, **(C)** fermentation temperature, and **(D)** fermentation time on the sensory scores of plant-based lotus seed yogurt. Different letters indicate significant differences (*p* < 0.05).

The single-factor experimental results showed that the highest sensory scores were achieved at a glucose concentration of 12%, inoculum size of 4%, fermentation temperature of 38 °C, and fermentation time of 8 h (Figure 2). These conditions were selected as the center points for response surface methodology optimization.

**Figure 3:**
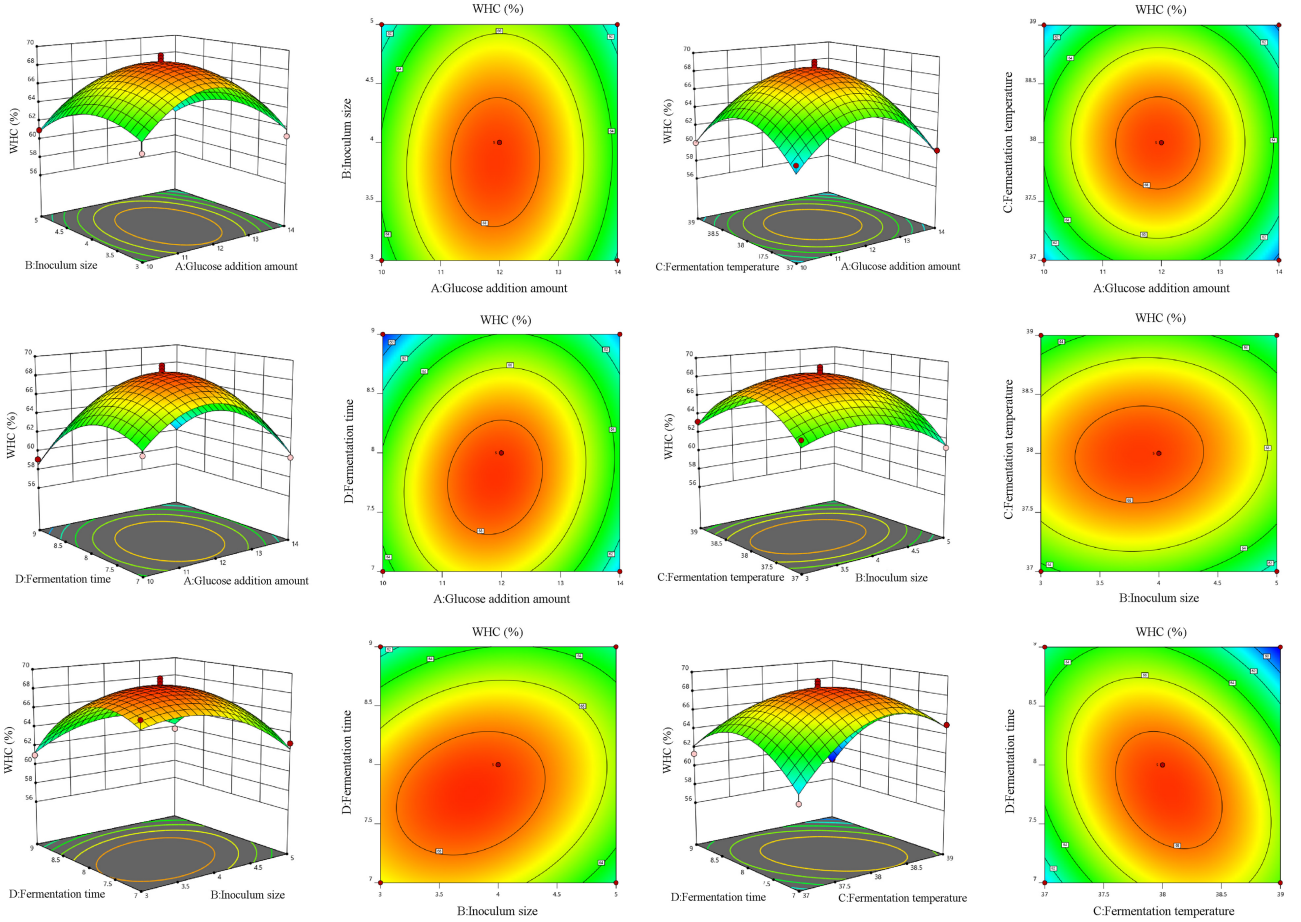
Response surface plots and corresponding contour plots showing the interactive effects of fermentation variables on the WHC of plant-based lotus seed yogurt. Different letters indicate significant differences (*p* < 0.05).

**Figure 4:**
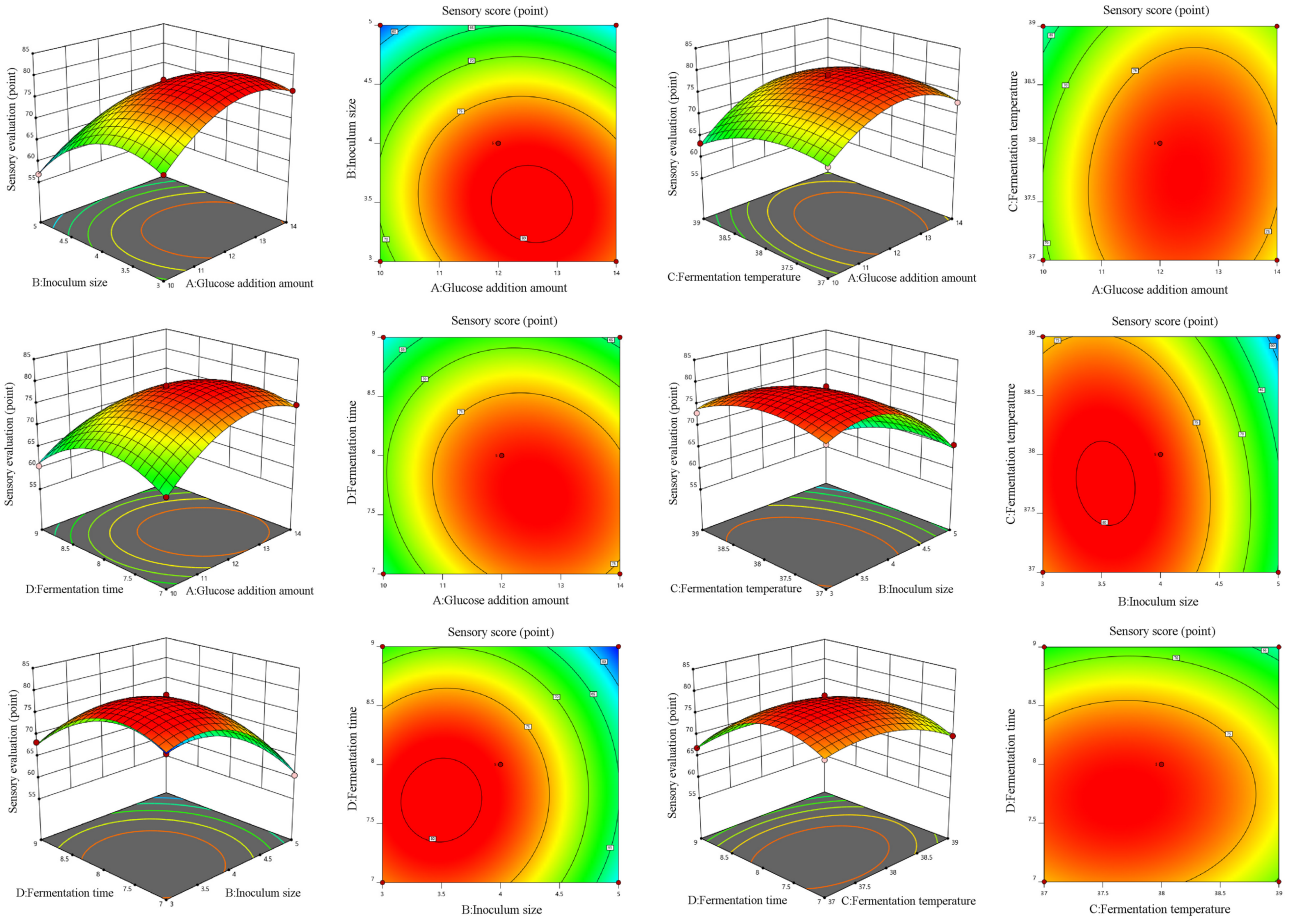
Response surface plots and corresponding contour plots illustrating the interactive effects of fermentation variables on the sensory evaluation scores of plant-based lotus seed yogurt.

A four-factor, three-level Box-Behnken design was applied to optimize glucose addition (A), inoculum size (B), temperature (C), and time (D), with WHC (Y1) and sensory score (Y_2_) as responses (Table 6). Both models were highly significant (*p* < 0.01) with non-significant lack of fit (*p* > 0.05). High coefficients of determination (*R*^2^=0.9679 and 0.9970), the adjusted $R_{adj}^{2}$ values (0.9357–0.9940), and low coefficients of variation (*CV*) values (1.33% and 0.8226%) confirmed a good fit and high reliability of the model. Based on the *p*-values, the linear terms B and D, the interaction terms BD and CD, and the quadratic terms A^2^, B^2^, C^2^, and D^2^ have a highly significant influence on WHC (*p*<*0.01*). The interaction term AD has a significant effect on WHC (*p*<*0.05*) (Table 7; Figure 3). For sensory evaluation, the linear terms A, B, C, and D, the interaction terms AB, AC, AD, and BC, and the quadratic terms A^2^, B^2^, C^2^, and D^2^ are highly significant (*p*<*0.01*), while the interaction term BD is significant (*p*<*0.05*) (Table 8; Figure 4). The remaining terms do not show a significant influence on either WHC or sensory evaluation.


$$\begin{array}{l} Y_{1}=68.61-0.2825A-0.7417B-0.0158C-1.29D+0.3250AB\\ +0.0550AC+1.08AD+0.3500BC+1.28BD-2.08CD \end{array}$$

$$\begin{array}{l} -4.75A^{2}-2.26B^{2}-3.97C^{2}-3.28D^{2} \end{array}$$ (2)

$$\begin{array}{l} Y_{2}=78.58+2.92A-6.78B-2.22C-3.58D-1.27AB\\ +0.8750AC-1.80AD-1.17BC+0.6500BD+0.3500CD \end{array}$$

$$\begin{array}{l} -6.22A^{2}-6.97B^{2}-3.42C^{2}-6.49D^{2} \end{array}$$ (3)

**Table 6:** Response surface experiment design and results.

Run	A	B	C	D	Y_1_	Y_2_
1	12	5	37	8	61.07	65.60
2	12	4	38	8	68.99	78.10
3	12	4	37	7	59.69	74.60
4	10	4	39	8	60.04	63.33
5	14	5	38	8	61.47	60.20
6	14	4	39	8	59.14	70.58
7	12	5	38	7	62.93	60.55
8	14	3	38	8	61.05	76.70
9	12	4	38	8	68.11	76.60
10	12	4	39	7	65.02	69.83
11	10	4	37	8	61.08	69.00
12	12	3	38	9	61.00	68.27
13	12	4	38	8	69.27	76.40
14	14	4	37	8	59.96	72.80
15	10	4	38	9	59.10	60.45
16	14	4	38	9	60.74	63.00
17	12	4	37	9	61.32	67.00
18	12	4	39	9	58.34	63.58
19	12	4	38	8	68.66	77.10
20	12	5	39	8	61.43	60.17
21	12	4	38	8	68.01	76.70
22	12	3	38	7	67.25	76.09
23	12	3	37	8	64.22	75.90
24	10	3	38	8	61.86	68.20
25	12	5	38	9	61.78	55.36
26	10	4	38	7	62.77	65.00
27	12	3	39	8	63.18	72.08
28	10	5	38	8	60.98	56.90
29	14	4	38	7	60.08	74.18

**Table 7:** Results of the analysis of variance for the regression model using WHC as the evaluation indicator.

Source	Sum of squares	DF	Mean square	F-Value	*p*-Value
Model	294.06	14	21.00	30.11	< 0.0001
A	0.9577	1	0.9577	1.37	0.2609
B	6.60	1	6.60	9.46	0.0082
C	0.0030	1	0.0030	0.0043	0.9486
D	19.92	1	19.92	28.55	0.0001
AB	0.4225	1	0.4225	0.6057	0.4494
AC	0.0121	1	0.0121	0.0173	0.8971
AD	4.69	1	4.69	6.72	0.0213
BC	0.4900	1	0.4900	0.7024	0.4160
BD	6.50	1	6.50	9.32	0.0086
CD	17.26	1	17.26	24.75	0.0002
A^2^	146.32	1	146.32	209.75	< 0.0001
B^2^	33.00	1	33.00	47.31	< 0.0001
C^2^	102.33	1	102.33	146.70	< 0.0001
D^2^	69.81	1	69.81	100.08	< 0.0001
Residual	9.77	14	0.6976		
Lack of fit	8.57	10	0.8574	2.88	0.1602
Pure error	1.19	4	0.2981		
Total	303.82	28			

Significant (*p* < 0.05), R^2^ = 0.9679, Adjusted R^2^ = 0.9357.

**Table 8:** Results of the analysis of variance for the regression model using sensory evaluation as the evaluation index.

Source	Sum of squares	DF	Mean square	F-Value	*p*-Value
Model	1511.13	14	107.94	334.84	<0.0001
A	102.08	1	102.08	316.68	<0.0001
B	552.16	1	552.16	1712.89	<0.0001
C	58.96	1	58.96	182.91	<0.0001
D	154.08	1	154.08	477.99	<0.0001
AB	6.50	1	6.50	20.17	0.0005
AC	3.06	1	3.06	9.50	0.0081
AD	12.96	1	12.96	40.20	<0.0001
BC	5.52	1	5.52	17.13	0.0010
BD	1.69	1	1.69	5.24	0.0381
CD	0.4900	1	0.4900	1.52	0.2379
A^2^	250.88	1	250.88	778.28	<0.0001
B^2^	315.04	1	315.04	977.31	<0.0001
C^2^	75.83	1	75.83	235.24	<0.0001
D^2^	273.56	1	273.56	848.63	<0.0001
Residual	4.51	14	0.3224		
Lack of fit	3.97	10	0.3965	2.89	0.1587
Pure error	0.5480	4	0.1370		
Total	1515.65	28			

Significant (*p* < 0.05), R^2^ = 0.9970, Adjusted R^2^ = 0.9940.

**Figure 5:**
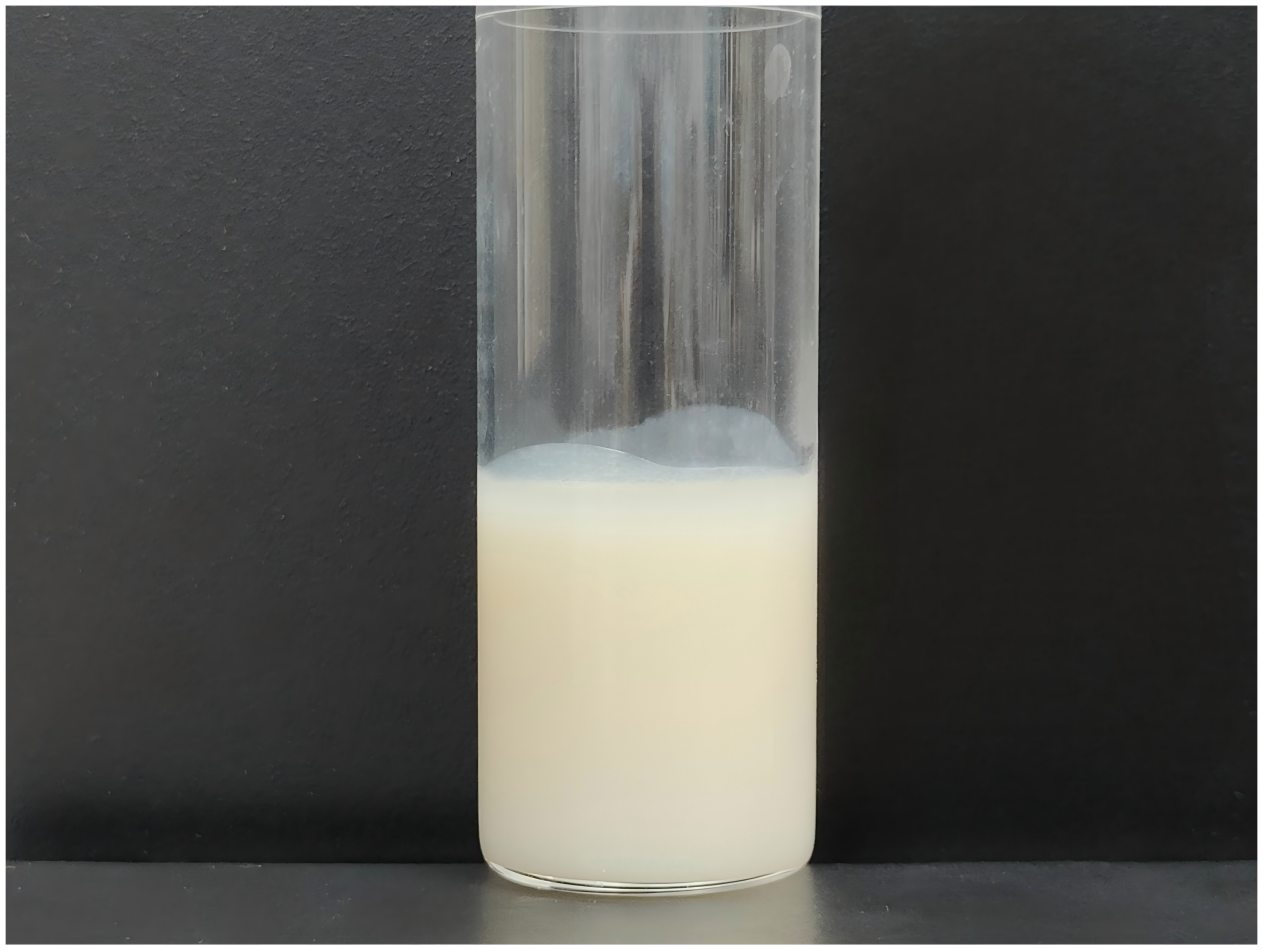
Representative photograph of the plant-based lotus seed yogurt produced under optimized fermentation conditions.

The predicted optimal conditions differed slightly for maximizing WHC and sensory score. Considering practical feasibility, final conditions were set at 12.2% glucose, 3.7% inoculum, 38 °C, and 7.5 h. Validation experiments yielded a WHC of 68.57% and a sensory score of 80.9, closely matching predictions. The resulting yogurt exhibited a uniform structure, smooth mouthfeel, balanced acidity, and characteristic lotus seed aroma (Figure 5).

### 3.3 Analysis of nutritional profile and physicochemical properties of lotus seed-based vegan yogurt

The lotus seed-based yogurt contained lower protein but significantly higher dietary fiber and carbohydrates compared to dairy yogurt (*p* < 0.05), with very low fat content (0.25 ± 0.01 g/100 g) and reduced energy value (272.47 ± 7.71 kJ/100 g) (Table 9). The lotus seed-based yogurt exhibited viable probiotic counts comparable to those of commercial dairy yogurt, and no *E. coli* was detected in either product. It also showed lower acidity (pH 4.01 ± 0.03) but significantly higher WHC (68.57% ± 0.15%) than dairy yogurt, along with higher viscosity (1,447.67 ± 4.29 mPa·s).

**Table 9:** Comparison of nutritional components between plant-based lotus seed yogurt and commercially available dairy-based yogurt.

Nutritional components	Plant-based lotus seed yogurt	Commercially available yogurt
Energy (kJ/100g)	272.47 ± 7.71^b^	379.83 ± 0.76^a^
Starch (g/100g)	3.53 ± 0.04^a^	3.14 ± 0.03^b^
Moisture (g/100g)	81.00 ± 0.26^b^	83.20 ± 0.11^a^
Fat (g/100g)	0.25 ± 0.01^b^	3.48 ± 0.05^a^
Protein (g/100g)	2.33 ± 0.013^b^	2.76 ± 0.012^a^
Dietary fiber (g/100g)	1.02 ± 0.06^a^	0.85 ± 0.06^b^
Carbohydrates (g/100g)	13.50 ± 0.44^a^	11.63 ± 0.12^b^

Values with different superscript letters are significantly different (*p*<*0.05*).

**Figure 6:**
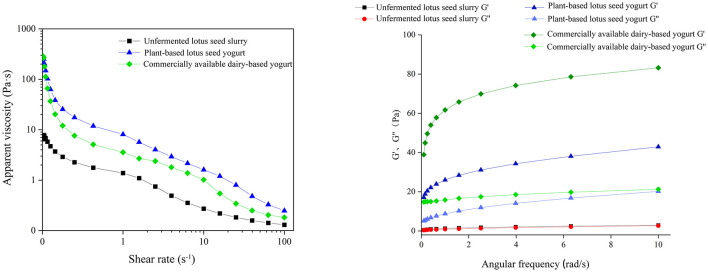
Comparative rheological properties of unfermented lotus seed slurry, plant-based lotus seed yogurt, and commercially available dairy yogurt. **(A)** apparent viscosity as a function of shear rate. **(B)** storage modulus (G′) and loss modulus (G″) as a function of angular frequency.

Colorimetric analysis revealed that the lotus seed–based yogurt had a warm ivory color, with L^*^ value of 65.57 ± 0.08, a^*^ value of −22.85±0.06, and b^*^ value of 5.85 ± 0.20 (Table 10). Rheological analysis indicated that all tested samples exhibited shear-thinning behavior (Figure 6A). The lotus seed-based yogurt displayed higher apparent viscosity than dairy yogurt, and fermentation significantly increased the viscosity compared to the unfermented lotus seed slurry. Frequency sweep analysis confirmed solid-like gel behavior after fermentation, with storage modulus (G′) consistently exceeding loss modulus (G″) (Figure 6B). In contrast, the unfermented lotus seed slurry showed nearly overlapping G′ and G″ curves.

**Table 10:** Comparison of physicochemical properties between plant-based lotus seed yogurt and commercially available dairy-based yogurt.

Index	Plant-based lotus seed yogurt	Commercially available yogurt
Probiotics (10^6^CFU/g)	133.33 ± 7.23^b^	160.67 ± 4.16^a^
*Escherichia coli*	ND	ND
Acidity(°T)	46.07 ± 0.12^b^	80.67 ± 0.15^a^
pH	4.01 ± 0.03^b^	4.17 ± 0.04^a^
WHC (%)	68.57 ± 0.15^a^	56.65 ± 0.31^b^
Viscosity (mPa·s)	1447.67 ± 4.29^a^	1410.07 ± 2.75^b^
L^*^	65.57 ± 0.08^b^	83.6 ± 0.06^a^
a^*^	−22.85 ± 0.06^b^	−16.00 ± 0.04^a^
b^*^	5.85 ± 0.20^a^	4.68 ± 0.03^b^

ND indicates not detected; Values with different superscript letters are significantly different (*p* < 0.05).

**Figure 7:**
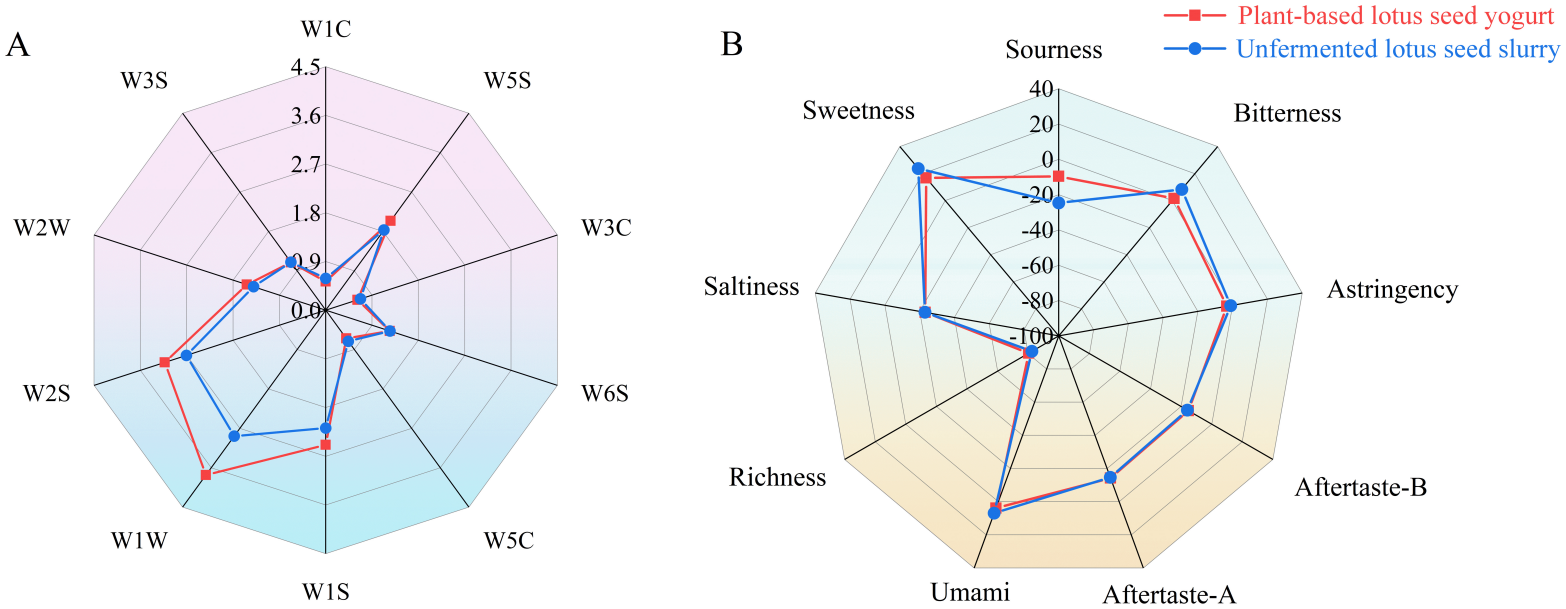
Comparison of odor and taste profiles of plant-based lotus seed yogurt before and after fermentation: **(A)** Electronic nose odor profile and **(B)** Electronic tongue taste profile.

The flavor evolution during fermentation was monitored using an electronic nose (Figure 7A). During fermentation, increased sensor responses associated with alcohols, aldehydes, and ketones were observed. The combined response intensity of the W1-series sensors (W1W, W1S) was significantly higher than that of the W2S sensor (*p* < 0.05). Electronic tongue analysis revealed an increase in sourness and a decrease in sweetness after fermentation (Figure 7B). Additionally, noticeable changes in bitterness and umami responses were detected.

**Figure 8:**
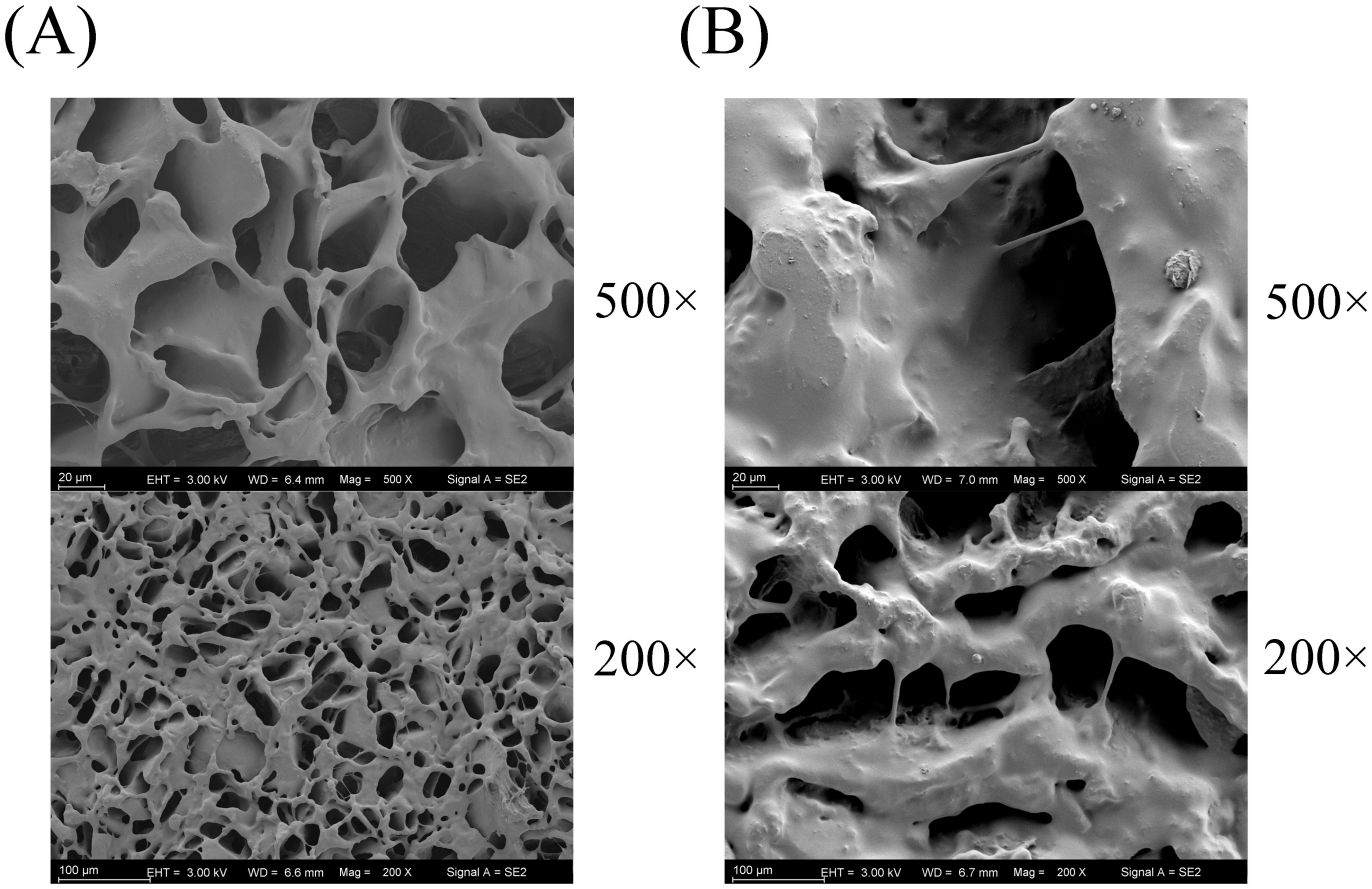
Microstructural characteristics of lotus seed matrices before and after fermentation. **(A)** unfermented lotus seed slurry. **(B)** plant-based lotus seed yogurt.

Significant microstructural changes were observed when comparing the scanning electron microscopy (SEM) images of unfermented lotus seed slurry and plant-based lotus seed yogurt (Figure 8). The images revealed a transition from a loose, porous structure in the unfermented slurry to a compact, homogeneous gel network in the yogurt after fermentation.

**Figure 9:**
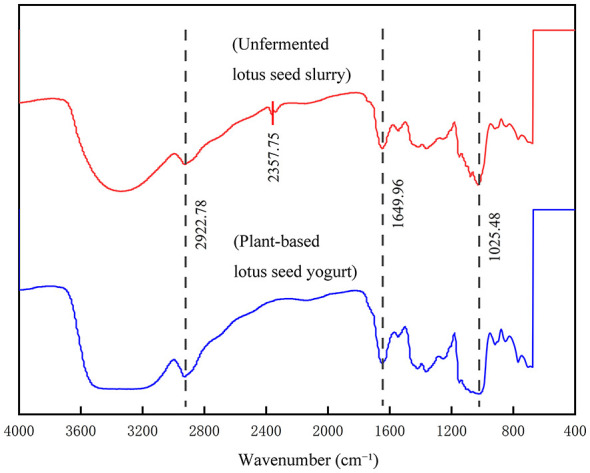
Fourier transform infrared (FTIR) spectra of plant-based lotus seed yogurt before and after fermentation.

The FTIR spectra (Figure 9) revealed that the primary absorption peaks remained at consistent wavenumbers before and after fermentation. A noticeable broadening and increased intensity of the band at 3300–3500 cm^−1^ were observed in the plant-based lotus seed yogurt.

**Figure 10:**
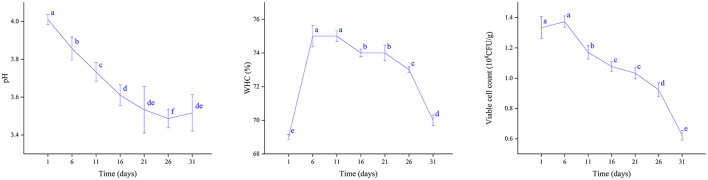
Changes in pH, WHC, and viable cell counts of plant-based lotus seed yogurt during refrigerated storage. Different letters indicate significant differences (*p* < 0.05).

The storage stability of the plant-based lotus seed yogurt was evaluated under refrigerated conditions at 4 °C (Figure 10). During storage, the pH gradually decreased to 3.49±0.05. The WHC increased during the first few days of storage, reaching a peak around day 6, before gradually declining. Probiotic counts increased from 133.33 ± 7.23 × 106 CFU/g to 137.33 ± 3.79 × 106 CFU/g during the initial stage of storage, followed by a significant decrease with prolonged storage.

### 3.4 Quantitative analysis of alkaloids in plant-based lotus seed yogurt

**Figure 11:**
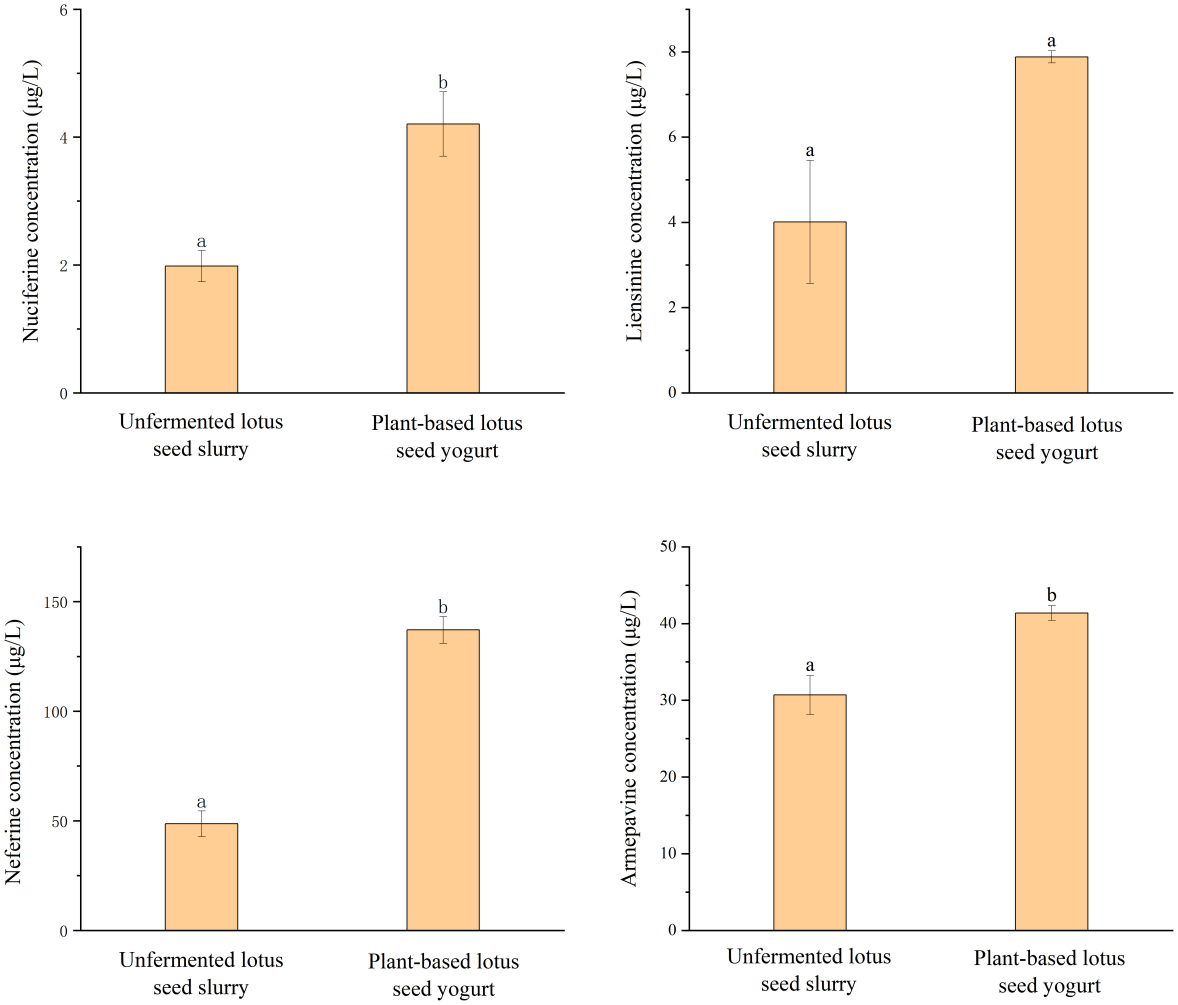
Comparison of alkaloid content between unfermented lotus seed slurry and plant-based lotus seed yogurt. Different letters indicate significant differences (*p* < 0.05).

Quantitative analysis confirmed the preservation of key sleep-related alkaloids in the plant-based lotus seed yogurt following fermentation (Figure 11). In the unfermented slurry, the concentrations of nuciferine, liensinine, neferine, and armepavine were measured at 1.983 μg/L, 4.010 μg/L, 48.666 μg/L, and 30.696 μg/L, respectively, while isococlaurine was not detected. After fermentation, these alkaloids were still preserved in the final product. The levels of nuciferine (4.205 μg/L), liensinine (7.883 μg/L), and armepavine (44.138 μg/L) remained present, with neferine also preserved at a measured concentration of 137.122 μg/L in the yogurt. Although the neferine value exceeded the method's upper quantification limit and warrants cautious interpretation, its detection confirms its presence after fermentation. Isococlaurine was not detected at any stage.

## 4 Discussion

Plant-based yogurt is gaining increasing market favor due to its health and environmental attributes (6). However, developing products from high-starch plants like lotus seeds still faces significant technological bottlenecks. Lotus seeds have an extremely high starch content (typically exceeding 60%), and their unique physicochemical properties lead to inherent difficulties during direct fermentation, including the formation of a fragile gel network, uneven texture, severe whey separation, and insufficient fermentation vigor (29, 30). These challenges have rendered it a “difficult-to-process” raw material for yogurt production. The innovation of this study lies in successfully developing a plant-based yogurt using lotus seeds as the primary matrix by optimizing the fermentation culture (*Streptococcus thermophilus, Lactobacillus plantarum*, and *Lactobacillus bulgaricus* in a 1:2:1 ratio). A comprehensive characterization of its nutritional composition, physicochemical and rheological properties, microstructure, and storage stability was conducted. This work provides a valuable strategy for addressing the technical barriers in applying similar plant resources to fermented foods and offers new insights for developing more diverse and localized plant-based yogurt products.

Nutritional analysis confirmed that the final product possesses a favorable profile for health-conscious consumers, being low in fat (0.25 ± 0.01 g/100 g) and calories (272.47 ± 7.71 kJ/100 g) while high in dietary fiber. This composition addresses growing dietary trends focused on weight management and metabolic health, offering a distinct advantage over conventional dairy yogurt for specific consumer groups. Physicochemical evaluation revealed a viscosity of 1,447.67 ± 4.29 mPa·s and a WHC of 68.57% for the lotus seed-based yogurt. These values are superior to those of typical dairy yogurts. The enhanced viscosity and water retention are indicative of a robust gel network, a critical factor for texture and shelf-life. This observation is consistent with the findings of Mäkinen et al. (31). The distinctive warm ivory color (L^*^ = 65.57 ± 0.08, a^*^ = −22.85 ± 0.06, b^*^ = 5.85 ± 0.20) is attributed to the inherent pigments of lotus seeds, differentiating the product visually from commercial dairy counterparts (32).

Rheological assessment confirmed the characteristic shear-thinning behavior of yogurt. Notably, the lotus seed-based yogurt exhibited a higher apparent viscosity than the commercial dairy control across the shear rate range, suggesting a richer, more ‘full-bodied' consistency. Dynamic frequency sweeps revealed that both products maintained a predominant solid-like character (G′ > G″). However, a more detailed comparison showed that the commercial dairy yogurt possessed a significantly higher storage modulus (G′) than the lotus seed-based yogurt, indicating that the casein-based matrix forms a stiffer and more robust gel network compared to the plant-protein/starch complex. While the lotus seed yogurt offers a “softer” gel texture, its structural integrity, as corroborated by SEM evidence showing a transition from a porous, discontinuous slurry to a dense, homogeneous gel after fermentation, is sufficient to resist structural breakdown during handling and storage. This structural integrity likely originates from synergistic interactions between lotus seed starch, proteins, and microbial exopolysaccharides produced during fermentation (33, 34).

The flavor and taste evolution during the fermentation of lotus seed-based yogurt, characterized by E-nose and E-tongue analyses, provides important insights into the formation of sensory quality in starch-rich plant-based fermented foods (35). The increased signals for alcohols, aldehydes, and ketones detected by the E-nose are a direct result of lactic acid bacteria metabolism. These volatile metabolites enrich the flavor complexity of the product, which aligns with the reported patterns of flavor formation in lactic acid bacteria-fermented plant-based beverages (36). The quantitative signal differences between the W1 and W2S sensor arrays confirm the dominant role of the characteristic lotus seed aroma throughout fermentation. The preservation of this core raw material aroma and its synergistic interaction with fermentation-derived volatile compounds are key to flavor optimization in plant-based fermented foods, consistent with findings in other fermented food systems where the raw material's aroma is retained and enhanced (37). The inversely correlated changes in sweetness and sourness revealed by the E-tongue are typical characteristics of plant-based fermentation systems, as has been well-documented in fermented legume products (38). Meanwhile, the alterations in bitterness and umami originate from the proteolytic activity of lactic acid bacteria, generating small-molecule peptides and free amino acids (39) as key taste-active compounds that significantly shape the product's aftertaste and overall flavor profile (40), ultimately enhancing the sensory quality of the plant-based lotus seed yogurt.

The FTIR spectra revealed that the primary absorption peaks remained at consistent wavenumbers before and after fermentation, suggesting that the covalent backbone and chemical integrity of the major components (e.g., proteins and polysaccharides) in lotus seed slurry were largely preserved (30). However, a noticeable broadening and increased intensity of the band at 3300–3500 cm^−1^ were observed in the plant-based lotus seed yogurt. This band corresponds to the stretching vibrations of -OH and -NH groups, and its intensification indicates the formation of a more extensive hydrogen-bonded network. This structural evolution is attributed to the acidification and protein denaturing during fermentation, which facilitates intermolecular cross-linking and contributes to the formation of the stable gel matrix of the yogurt (31).

The post-acidification observed during storage is commonly reported in fermented dairy and plant-based products and is attributed to the continued metabolic activity of residual lactic acid bacteria even under refrigerated conditions (41). This process contributes to the characteristic tangy flavor of yogurt, and the pH remained within an acceptable range throughout the storage period, indicating that the product retained good quality without excessive acidification. The initial increase in WHC is likely due to starch retrogradation, where amylose and amylopectin chains reassociate through hydrogen bonding, forming a more compact gel network that traps water more effectively (42). As storage continues, the gel network undergoes slow structural relaxation, eventually leading to syneresis and a progressive loss of water retention, a common phenomenon in starch-based gel systems (43).The initial increase in probiotic counts is likely due to continued growth using residual nutrients, while the subsequent decline reflects the combined effects of nutrient depletion, increased acidity, and cold stress during extended refrigerated storage (44).

In this study, HPLC-MS/MS analysis was also employed to characterize the alkaloid components in lotus seed-based yogurt. A key finding of this work is the significant fermentation-mediated enrichment of bioactive isoquinoline alkaloids native to lotus seeds, such as nuciferine and liensinine. We propose that this enrichment results from the biotransformative activity of the lactic acid bacteria consortium (45). Specifically, bacterial enzymes such as glycosidases may hydrolyze conjugated or glycosylated forms of these alkaloids, releasing free, bioaccessible monomers (46). Similar biotransformation mechanisms have been reported in fermented plant matrices, where lactic acid bacteria mediate the conversion of bound phenolic compounds and alkaloids into their free forms (46, 47). This positions fermentation not just as a processing step for texture and preservation, but as a targeted bioprocessing strategy to amplify the intrinsic functional properties of the plant matrix. It should be noted, however, that the detected concentration of neferine exceeded the upper limit of quantification of the analytical method used; therefore, the reported value should be interpreted as semi-quantitative. Nevertheless, the detection above this threshold qualitatively confirms its persistence after fermentation. Given the documented pharmacological activities of these alkaloids (e.g., sedative, anxiolytic) (13, 48), their enrichment following fermentation suggests the potential for functional retention. This compositional shift provides a basis for further investigation into the bioactivity of fermented lotus seed products. Nuciferine, for instance, has been shown to exert sedative and anxiolytic effects in animal models at oral doses of 50 and 100 mg/kg in a dose-dependent manner (13). It should be noted that the nuciferine concentrations detected in this study are substantially lower than the pharmacological doses used in animal studies, and the product is intended for regular dietary consumption rather than acute pharmacological intervention.

Despite these promising findings, this study has several limitations. The quantification of neferine was semi-quantitative due to concentrations exceeding the analytical range. The investigation was confined to *in vitro* analyses, lacking *in vivo* validation of bioavailability or functional activity. Additionally, microbiological safety assessment was limited to *E. coli*, without evaluation of other pathogens or probiotic viability during storage. Future work should address these gaps through improved quantification methods, *in vivo* studies, and expanded safety evaluations, alongside process scale-up to support industrial application.

## 5 Conclusion

In this study, we successfully established an optimized process for producing high-quality plant-based yogurt using lotus seeds as the raw material. The final product demonstrated distinct nutritional characteristics, including low fat, low calorie, and high dietary fiber content, aligning with modern demands for healthy diets. The uniform and dense gel network formed during fermentation was key to achieving the product's excellent WHC and favorable rheological properties. Notably, fermentation significantly increased the concentrations of multiple alkaloids associated with neuroregulation, providing a compositional basis for the potential development of functional plant-based foods with sleep-promoting properties.

Overall, this study offers a novel plant-based yogurt product that aligns with consumer demand for healthy and functional foods, while also promoting the high-value utilization of lotus seeds. In the context of the rapidly expanding plant-based food sector, this work introduces a novel application of lotus seeds—a traditional “food-medicine homology” ingredient—as a viable substrate for plant-based yogurt production. The innovative integration of fermentation optimization, quality evaluation, and bioactive alkaloid analysis provides a scientifically grounded framework for transforming underutilized agricultural resources into functional food products. This approach not only expands the diversity of plant-based yogurt offerings but also demonstrates a strategy for enhancing the functional value of plant-based matrices through targeted fermentation. By bridging traditional food heritage with modern food technology, this study offers a scalable and value-added solution that supports the sustainable development of the plant-based food industry.

Future research could focus on process scale-up, precise flavor modulation, and further validation of functional activities such as sleep promotion through *in vitro* or *in vivo* models.

## Data Availability

The original contributions presented in the study are included in the article/Supplementary material, further inquiries can be directed to the corresponding authors.
