# Supplementary material for: Development of lotus seed-based vegan yogurt: fermentation optimization, quality evaluation and analysis of key bioactive alkaloids
Source: Front Nutr. 2026 Apr 28;13:1825059. doi: 10.3389/fnut.2026.1825059 (PMC13160716; doi:10.3389/fnut.2026.1825059)
Supplement: Supplementary file 1 [file Table_1.pdf]

Supplementary Material

Supplementary Table 1. Comprehensive physicochemical properties of frozen fresh lotus seeds.

Indexes	Lotus seeds
Dietary fiber (g/100g)	2.99 ± 0.24
Carbohydrate (g/100g)	17.82 ± 1.20
Total polyphenol (mg/100g)	69 ± 4
pH*	6.95 ± 0.04

* Lotus seeds were grinded, filtered, and the resulting filtrate was analyzed for pH.
